# Sex-Related Differences in the Effects of Sleep Habits on Verbal and Visuospatial Working Memory

**DOI:** 10.3389/fpsyg.2016.01128

**Published:** 2016-07-28

**Authors:** Seishu Nakagawa, Hikaru Takeuchi, Yasuyuki Taki, Rui Nouchi, Atsushi Sekiguchi, Yuka Kotozaki, Carlos M. Miyauchi, Kunio Iizuka, Ryoichi Yokoyama, Takamitsu Shinada, Yuki Yamamoto, Sugiko Hanawa, Tsuyoshi Araki, Keiko Kunitoki, Yuko Sassa, Ryuta Kawashima

**Affiliations:** ^1^Department of Human Brain Science, Institute of Development, Aging and Cancer, Tohoku UniversitySendai, Japan; ^2^Department of Psychiatry, Tohoku Medical and Pharmaceutical UniversitySendai, Japan; ^3^Division of Developmental Cognitive Neuroscience, Institute of Development, Aging and Cancer, Tohoku UniversitySendai, Japan; ^4^Division of Medical Neuroimaging Analysis, Department of Community Medical Supports, Tohoku Medical Megabank Organization, Tohoku UniversitySendai, Japan; ^5^Department of Nuclear Medicine and Radiology, Institute of Development, Aging and Cancer, Tohoku UniversitySendai, Japan; ^6^Creative Interdisciplinary Research Division, Frontier Research Institute for Interdisciplinary Science, Tohoku UniversitySendai, Japan; ^7^Human and Social Response Research Division, International Research Institute of Disaster Science, Tohoku UniversitySendai, Japan; ^8^Smart Ageing International Research Center, Institute of Development, Aging and Cancer, Tohoku UniversitySendai, Japan; ^9^Department of Adult Mental Health, National Institute of Mental Health, National Center of Neurology and PsychiatryTokyo, Japan; ^10^Department of General Systems Studies, Graduate School of Arts and Sciences, The University of TokyoTokyo, Japan; ^11^Department of Psychiatry, Tohoku University Graduate School of MedicineSendai, Japan; ^12^School of Medicine, Kobe UniversityKobe, Japan; ^13^Japan Society for the Promotion of ScienceTokyo, Japan; ^14^ADVANTAGE Risk Management Co., LtdTokyo, Japan; ^15^Faculty of Medicine, Tohoku UniversitySendai, Japan; ^16^Advanced Brain Science, Institute of Development, Aging and Cancer, Tohoku UniversitySendai, Japan

**Keywords:** dream content remembering frequency (DCRF), nap duration, sleep duration, verbal working memory capacity (VWMC), visuospatial working memory capacity (VSWMC)

## Abstract

Poor sleep quality negatively affects memory performance, and working memory in particular. We investigated sleep habits related to sleep quality including sleep duration, daytime nap duration, nap frequency, and dream content recall frequency (DCRF). Declarative working memory can be subdivided into verbal working memory (VWM) and visuospatial working memory (VSWM). We hypothesized that sleep habits would have different effects on VWM and VSWM. To our knowledge, our study is the first to investigate differences between VWM and VSWM related to daytime nap duration, nap frequency, and DCRF. Furthermore, we tested the hypothesis that the effects of duration and frequency of daytime naps and DCRF on VWM and VSWM differed according to sex. We assessed 779 healthy right-handed individuals (434 males and 345 females; mean age: 20.7 ± 1.8 years) using a digit span forward and backward VWM task, a forward and backward VSWM task, and sleep habits scales. A correlation analysis was used to test the relationships between VWM capacity (VWMC) and VSWM capacity (VSWMC) scores and sleep duration, nap duration, nap frequency, and DCRF. Furthermore, multiple regression analyses were conducted to identify factors associated with VWMC and VSWMC scores and to identify sex-related differences. We found significant positive correlations between VSWMC and nap duration and DCRF, and between VWMC and sleep duration in all subjects. Furthermore, we found that working memory capacity (WMC) was positively correlated with nap duration in males and with sleep duration in females, and DCRF was positively correlated with VSWMC in females. Our finding of sex-related differences in the effects of sleep habits on WMC has not been reported previously. The associations between WMC and sleep habits differed according to sex because of differences in the underlying neural correlates of VWM and VSWM, and effectiveness of the sleep habits in males and females.

## Introduction

Poor sleep quality has a negative affect on memory performance, particularly working memory (Valdez et al., 2008). Considerable evidence suggests that sleep habits, such as sleep duration, daytime nap duration, nap frequency, and dream content recall, are related to the quality of sleep. Less than 7 h per night affects cognitive function (Banks and Dinges, 2007) and working memory (del Angel et al., 2015). Daytime naps increase alertness and cognitive performance (Milner and Cote, 2009) and, thus, improve learning and memory. Importantly, the benefits of a brief (5–15 min) nap are almost immediate but short lasting (1–3 h), and although longer naps (>30 min) may produce a short period of sleep inertia, cognitive performance is improved for several hours after waking (Lovato and Lack, 2010). Habitual napping improves alertness (Milner and Cote, 2009). Thus, the duration and frequency of daytime naps have an impact on memory performance. Furthermore, because one can memorize the contents of dreams, the act of recalling a dream must involve memory operations. Specifically, the recall of dream content may involve two steps: recalling the dream experiences and considering the dream content by associating it with personal memories (Nielsen and Stenstrom, 2005). The most important variable related to dream recall may be individual differences in memory ability (Cory and Ormiston, 1975). Dream content recall may be enhanced by increasing the capacity of short-term memory and imaginal life (Martinetti, 1985). Moreover, high dream content recall frequency (DCRF) is associated with higher visuospatial IQ (Butler and Watson, 1985) and absorption in imagery, according to the results of a subjective questionnaire (Schredl et al., 1997). However, several studies have been unable to replicate these findings or have reported contradictory results (Dumel et al., 2015).

Declarative working memory can be subdivided into verbal (VWM) and visuospatial (VSWM) working memory. VWM is used for speech, reading, and writing, whereas VSWM is used for spatial processing, drawing, and mathematics (Valdez et al., 2008). It is likely that sleep habits affect VWM and VSWM differently because their neural correlates differ considerably. VWM is lateralized in the left hemisphere, most notably in the frontal and parietal lobes, whereas VSWM is lateralized in the right hemisphere, particularly in the frontal and temporal cortices (Nagel et al., 2013). A reduction in daily sleep duration has been shown to decrease accuracy in VWM (Jiang et al., 2011) and VSWM (Kuriyama et al., 2008) tasks in healthy young adults. Moreover, a previous study in healthy young subjects found that VSWM function was better in subjects who had a nap than in those who did not (Lau et al., 2015). Based on these findings, we hypothesized that the duration and frequency of daytime naps and DCRF would have different effects on VWM and VSWM. To our knowledge, no previous study has investigated the effects of nap duration and frequency and DCRF on VWM and VSWM simultaneously. Thus, we investigated the relationships between daily sleep habits and working memory.

A previous review of studies investigating sex differences in working memory found that males performed better than females on tasks that required transformations in VSWM, whereas females performed better on tasks that used phonological and semantic information (Halpern, 1997). Furthermore, a study in university undergraduates found that males performed better than females on a high cognitive load spatial working memory task (Lejbak et al., 2011). Based on these findings, we further hypothesized that the associations between VWM and VSWM and sleep habits would differ according to sex. Thus, we investigated the associations among working memory and sleep habits according to sex.

## Materials and Methods

### Subjects

Our study included 779 (434 males and 345 females; mean age, 20.7 ± 1.8 years) healthy right-handed individuals with normal vision who were part of an ongoing project investigating associations between brain imaging, cognitive function, aging, genetics, and daily habits (Takeuchi et al., 2010, 2011a). Our findings will be made available to researchers investigating other psychological and behavioral phenomena, such as chronic fatigue and fatigability. All subjects were university, college, or postgraduate students who had graduated from their respective institutions within 1 year of the initiation of our experiment. Japanese schools typically provide annual health checkups. The medical checkup for first-year students is performed in early April, before classes start. The screening items gather general information and include height and weight measurements, a blood/urine test, and a chest X-ray^1^. After participants have completed the health examination, the Health Administration Center is available to help with retesting, explaining the results, providing health-related guidance and hospital referrals, and so on. As described previously (Takeuchi et al., 2015b), we also distributed self-report questionnaires to potential subjects to assess their history of psychiatric and physical illness as well as their recent drug use. The questionnaires required subjects to provide a detailed list of any recent drugs used. The assessments performed during and after recruitment were voluntary and self-reported. As we described in our previous study (Takeuchi et al., 2015a), we did not control the menstrual cycle of the female participants. Thus, the associations reported by the female participants in this study represent the average of the associations experienced across the various stages of the menstrual cycle. During the recruitment procedure, the exclusion criteria, including a restriction on individuals with physical or mental health conditions, were explained to all subjects. Potential subjects were reminded of the exclusion criteria after the preliminary contact to prevent individuals who should have been excluded from the study from arriving at the laboratory to participate. Subjects who met the exclusion criteria and arrived to participate in the experiment were asked to return home. Accordingly, none of the subjects included in our study had a history of neurological or psychiatric illnesses. As we described in our previous study (Takeuchi et al., 2015a), subjects were instructed to get sufficient sleep, maintain their physical condition, eat an adequate breakfast, and consume their normal amounts of caffeinated food and drink on the day of cognitive testing. Additionally, subjects were instructed to avoid alcohol the night before the assessment.

Written informed consent was obtained from each subject prior to participation in the study in accordance with the Declaration of Helsinki (1991), and the study protocol was approved by the Ethics Committee of Tohoku University.

### Psychological Outcome Measures

#### Assessment of VWM Capacity (VWMC)

Computerized forward and backward digit span tests were used to assess VWM capacity (VWMC). These tests were used to adjust for the effect of individual psychometric measures of VWMC on brain activities during VWM processes. Subjects were asked to view a progressively increasing number of random digits, starting at two digits, visually presented at a rate of one digit/s on a computer screen. They were then asked to repeat the sequence by pressing numbered buttons on the screen in the order presented (digit span forward) or in the reverse order (digit span backward). Three sequences were presented at each level until the participants responded incorrectly to all three sequences, at which point the task was ended. The score of each test was the sum of the number of digits correctly repeated in the digit span forward and backward tasks (Takeuchi et al., 2011a).

#### Assessment of VSWM Capacity (VSWMC)

In the VSWM task, circles were presented one by one at a rate of one/s in a four-by-four grid-like interface. Participants were required to remember the location and order of the stimuli. After the stimuli were presented, participants were instructed to indicate the location and order presented (forward VSWM task) or reverse order (backward VSWM task) of the stimuli by clicking the grid-like interface on a computer screen using a mouse. The number of items to be remembered started with two items and progressively increased. Three sequences were presented at each level until the participants responded incorrectly to all three sequences, at which point the task was ended. The score of each test was the sum of the number of items correctly repeated in the forward and backward VSWM tasks (Takeuchi et al., 2011b).

#### Assessment of Sleep Habits

We used the Japanese version of the Sleep Habits Scale developed at the Tokyo Metropolitan Institute of Medical Science (Ishihara et al., 1986). The questionnaire is a widely used self-report measure of individual sleep habits. The responses are compiled into a single score, in which higher scores indicate longer duration and greater frequency of the respective measures. Both the original and Japanese versions of the Sleep Habits Scale have high reliability (Ishihara et al., 1986; Anderson et al., 1991). We assessed daily sleep and nap duration, nap frequency, and DCRF. Daily sleep duration was assessed using a 15-point scale ranging from <4 h (1) to >10 h (15) in 30 min increments. Daily nap duration was assessed using a four-point rating scale (1. ≤15 min, 2. >15 ≤ 30 min, 3. >30 ≤ 60 min, 4. >60 min), and nap frequency was measured using a four-point rating scale (1. seldom, 2. sometimes, 3. often, 4. habitual). DCRF was assessed using a six-point rating scale (1. seldom, 2. rarely, 3. a few, 4. sometimes, 5. often, 6. always).

### Behavioral Data Analyses

All statistical tests were conducted using the IBM Statistical Package for the Social Sciences 22.0 (SPSS, IBM Corp., Armonk, NY, USA). Sex-related differences in age, VWMC and VSWMC scores, and sleep habits were tested using Mann–Whitney *U* tests with a Bonferroni correction (*P* < 0.05/7 = 0.0071) for multiple comparisons to determine statistical significance. Spearman correlation coefficients were used to test relationships between VWMC and VSWMC scores and sleep habits. *P*-values <0.0071 (0.05/7, two-tailed corrected using the Bonferroni method) were deemed to indicate statistical significance.

To identify the factors associated with VWMC and VSWMC scores, we conducted a stepwise regression analysis using VWMC and VSWMC scores as dependent variables and age, sex, sleep duration, nap duration, nap frequency, and DCRF as independent variables. *P*-values <0.05 (two-sided probability) were deemed to indicate statistical significance. Furthermore, we performed separate step-wise regression analyses in males and females, using the same method, to investigate sex-related differences in VWMC and VSWMC using VWMC and VSWMC scores as dependent variables and age, sleep duration, nap duration, nap frequency, and DCRF as independent variables.

Structural equation modeling (SEM) is useful for the assessment of mediation because it offers several alternatives for exploring mediation effects (Preacher and Hayes, 2004). Thus, we used SEM to test our hypothesis that the interactions between VWMC and VSWMC are mediated by individual differences in sleep duration, nap duration, and DCRF. All factors that made significant independent contributions to the VWMC and VSWMC scores were entered into a linear structural equation system using analysis of moment structures (AMOS 18) to explore the interrelationships of working memory capacity (WMC) with sleep duration, nap duration, and DCRF. We constructed a model in which VWMC and VSWMC interactions were mediated by sleep duration, nap duration, and DCRF based on the results of the multiple regression analysis. We also constructed a model in which interactions between VWMC and VSWMC were mediated by the aforementioned sleep habits, and sex (male and female) was treated as a covariate.

## Results

**FIGURE 1:**
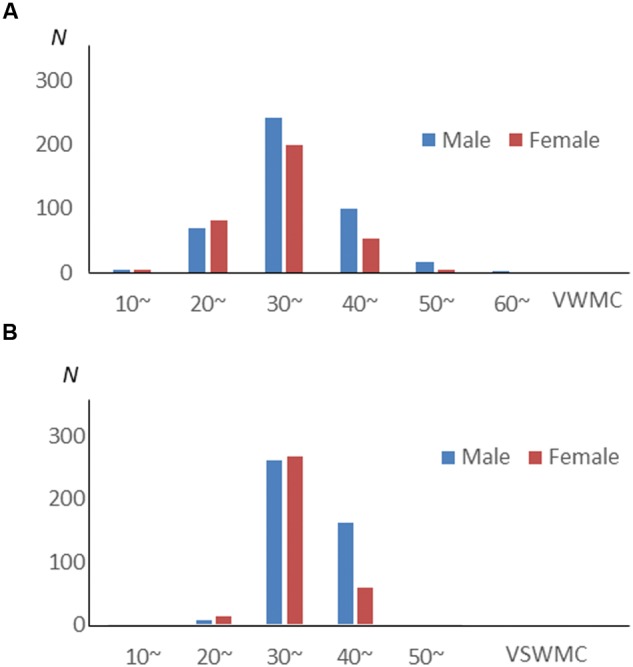
**VWMC and VSWMC score distributions according to sex.** Histograms showing the distributions of verbal working memory capacity (VWMC, **A**) and visuospatial working memory capacity (VSWMC, **B**) scores for males (Blue bars) and females (Red bars).

**FIGURE 2:**
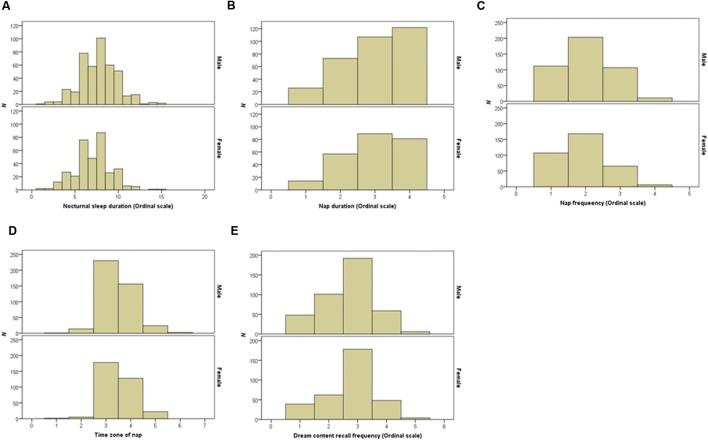
**Distributions of nocturnal sleep duration, nap duration, nap frequency, time zone of nap, and dream recall frequency in both sexes.** Histograms show the distributions of nocturnal sleep duration **(A)**, nap duration **(B)**, nap frequency **(C)**, time zone of nap **(D)**, and dream recall frequency **(E)** in both sexes.

Verbal working memory capacity and VSWMC score distributions according to sex are shown in **Figure 1**. The histograms in **Figure 2** show the distributions of sleep habits in both sexes. The VWMC and VSWMC scores and sleep duration and nap frequency values were significantly greater in males than in females (Mann–Whitney *U* tests with Bonferroni correction, *P* < 0.05/7 = 0.0071; **Table 1**). We found significant positive correlations between VSWMC and VWMC scores and nap duration (*P* < 0.05, two-tailed corrected using the Bonferroni method; **Table 2**).

**Table 1:** Age, VWMC, and VSWMC scores, and sleep habits according to sex.

	All subjects		Males		Females		*P*-value	*Z*
					
Variable	Mean	*SD*	Mean	*SD*	Mean	*SD*		
Age	20.7	1.8	20.8	2.0	20.6	1.7	0.42	0.82
VWMC	35.7	7.1	36.7	7.2	34.5	6.8	<0.0001^∗^	3.99
VSWMC	28.3	4.3	29.3	4.1	27.1	4.2	<0.0001^∗^	7.25
Sleep duration	7.5	2.2	7.7	2.2	7.1	2.1	<0.0001^∗^	4.13
Nap duration	3.0	0.9	3.0	1.0	3.0	0.9	0.752	0.32
Nap frequency	2.0	0.8	2.0	0.8	1.9	0.7	0.024	2.26
DCRF	2.7	0.9	2.7	0.9	2.8	0.9	0.283	1.07


*^∗^*P* < 0.05/7 = 0.0071 (Mann–Whitney *U* test with Bonferroni correction). DCRF, dream content recall frequency; SD, standard deviation; VWMC, verbal working memory capacity; VSWMC, visuospatial working memory capacity.*

**Table 2:** Correlations between age, VWMC, and VSWMC scores, and sleep habits.

	Age	VWMC	VSWMC	Sleep duration	Nap duration	Nap frequency
Age	–					
VWMC	0.204	–				
VSWMC	0.007	0.308^∗^	–			
Sleep duration	0.048	0.090	0.089	–		
Nap duration	-0.152^∗^	0.049	0.164^∗^	0.124^∗^	–	
Nap frequency	-0.081	-0.02	-0.031	0.035	0.116^∗^	–
DCRF	-0.018	0.019	0.067	-0.016	0.071	0.066


*^∗^*P* < 0.05/7 = 0.0071 (Spearman correlation coefficients corrected using the Bonferroni method). DCRF, dream content recall frequency; VWMC, verbal working memory capacity; VSWMC, visuospatial working memory capacity.*

The VWMC and VSWMC scores were used as dependent variables in a stepwise regression analysis (Entry; *P* < 0.05, Removal; *P* < 0.10) to identify the sleep variables independently associated with WMC (**Table 3**). The regression analysis revealed that nap duration and DCRF were significantly associated with VSWMC in all subjects, whereas sleep duration was significantly associated with VWMC. Nap duration was significantly correlated with VWMC and VSWMC in males, whereas sleep duration was significantly correlated with VWMC and VSWMC in females. Moreover, DCRF was significantly associated with VSWMC in females.

**Table 3:** Factors associated with VWMC and VSWMC: Multiple stepwise regression analyses in all subjects, males, and females.

Subjects	Dependent variables	Independent variables	*R*	Adjusted *R*^2^	*F*	β
All	VWMC	Sex	0.186	0.031	9.51ˆ***	0.144
		Sleep duration				0.103
	VSWMC	Sex	0.315	0.094	19.49ˆ***	0.236
		Nap duration				0.162
		DCRF				0.119
Males	VWMC	Nap duration	0.113	0.009	3.90ˆ*	0.113
	VSWMC	Nap duration	0.199	0.036	12.55ˆ***	0.199
Females	VWMC	Sleep duration	0.205	0.038	9.95ˆ**	0.205
	VSWMC	Sleep duration	0.256	0.057	7.91ˆ***	0.204
		DCRF				0.184


*^∗^*P* < 0.05, ^∗∗^*P* < 0.01, ^∗∗∗^*P* < 0.001. DCRF, dream content recall frequency; VWMC, verbal working memory capacity; VSWMC, visuospatial working memory capacity.*

**FIGURE 3:**
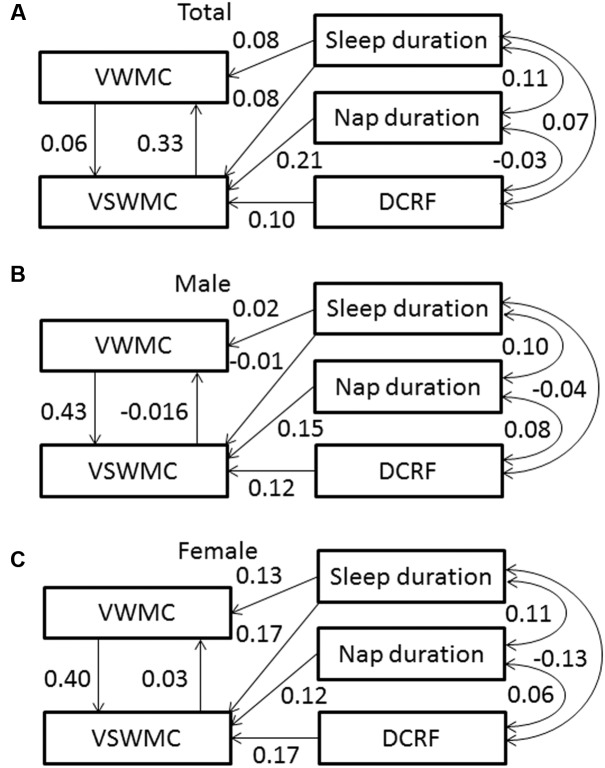
**Structural equation model showing associations between VWMC, VSWMC, sleep duration, nap duration, and DCRF in total **(A)**, male **(B)**, and female **(C)** subjects.** Sleep duration increased VWMC and VSWMC, whereas nap duration and DCRF increased only VSWMC. VWMC and VSWMC interact with each other. The one-headed arrows indicate the direction of the observed effect. The numbers on the arrows are standardized regression coefficients. Error components are omitted for simplicity. DCRF, dream content recall frequency; VSWMC, visuospatial working memory capacity; VWMC, verbal working memory capacity.

**Figure 3A** shows the SEM of interactions among VWMC and VSWMC mediated by individual differences in sleep duration, nap duration, and DCRF. The model provided a good fit for the data (goodness of fit index [GFI] = 0.997, adjusted goodness of fit index [AGFI] = 0.984, comparative fit index [CFI] = 0.980, and root mean square error of approximation [RMSEA] = 0.045). **Figure 3B** (male) and **Figure 3C** (female) show the SEMs of interactions between VWMC and VSWMC scores mediated by individual differences in sleep duration, nap duration, and DCRF. The model including sex as a covariate also provided a good fit for the data [GFI = 0.997, AGFI = 0.962, comparative fit index (CFI) = 0.987, and RMSEA = 0.037].

## Discussion

To our knowledge, our study is the first to investigate associations between WMC (VWMC and VSWMC) and sleep habits according to sex. We found that nap duration was correlated with VSWMC in all subjects, and we identified sex-related differences in the associations among VWMC, VSWMC, and sleep habits (i.e., nap duration in males and sleep duration and DCRF in females). Our finding of sex-related differences in the effect of sleep habits on WMC has not been reported previously.

Our finding that nap duration was significantly related to VWMC and VSWMC in males is in partial agreement with a previous study in which females showed greater improvement than males in declarative memory tasks, regardless of whether they had had a nap (Backhaus and Junghanns, 2006). Moreover, a previous study found that males performed significantly better on a memory consolidation task after a nap, whereas females did so only in the mid-luteal phase of their menstrual cycle (Genzel et al., 2012). These findings may explain the sex-related differences in our study. Furthermore, the accumulated effect of naps may be related to frequency and duration. Thus, the fact that the males in our study napped more frequently than females may account for the effect on WMC. Our finding that DCRF was significantly correlated with VSWMC in females is consistent with a previous meta-analysis, which found that females tended to recall their dreams more often than males (Schredl and Reinhard, 2008).

Our finding that DCRF was associated with VSWMC, but not VWMV, is supported by several lines of evidence. First, dream recall can be enhanced by increasing the capacity of short-term memory (Martinetti, 1985), and higher DCRF is associated with higher visuospatial IQ (Butler and Watson, 1985). Furthermore, DCRF is thought to be associated with VSWMC because the dreamer often portrays wishes, conflicts, or current problems in terms of visuospatial representations (Katz, 2005). Interestingly, the cortical region responsible for visuospatial integration, which includes the inferior parietal cortex, is selectively activated during REM sleep (Hobson, 2004; Pace-Schott, 2011). Thus, DCRF may be associated with improved VSWMC.

Our finding of a significant positive association between nap duration and VSWMC, but not VWMC, in all subjects is in partial agreement with that of a previous study in healthy young subjects showing that naps improved VSWMC (N-back task; Lau et al., 2015). Spatial working memory is impaired by acute (Olver et al., 2015) and chronic stress (Conrad, 2010) and the slow-wave sleep achieved during long naps has a stress-reducing effect (Faraut et al., 2013). Furthermore, previous studies have found that subjective feelings of anxiety decreased among participants in a nap group compared with a no-nap group (Takeyama et al., 2005), and that anxiety selectively disrupted the accuracy of spatial but not VWM performance (Shackman et al., 2006). Interestingly, brief meditation training sessions designed to maintain a relaxed state of mind, which is similar to napping, improved visuospatial processing (Zeidan et al., 2010), suggesting that longer naps may improve VSWMC by reducing stress and anxiety. It is important to explain why nap duration is correlated with better working memory function. Given the higher probability of being awakened from slow-wave sleep as nap duration increases, we would expect that the risk of sleep inertia (which transiently impairs working memory functions) would also increase as a function of nap duration (Tassi and Muzet, 2000). However, the duration of sleep inertia rarely exceeds 30 min in the absence of nocturnal sleep deprivation (Tassi and Muzet, 2000). VWM and VSWM were seldom measured during sleep inertia.

It is important to note that, in most time zones, naps occurred from noon to 3 p.m. (value 3, 53.5%) and from 3 p.m. to 6 p.m. (value 4, 37.3%). These data differ from the results of a previous study with university students in Madrid, Spain, which found that most naps (90%) took place later, after lunch or in the early afternoon (Vela-Bueno et al., 2008). This difference might be due to the fact that, unlike the situation in Japan, in the Mediterranean, including Spain, taking an afternoon nap forms part of the culture and is considered to be a healthy habit (Masa et al., 2006).

Our finding that sleep duration was positively correlated with VWMC in all subjects is consistent with those of previous studies showing a positive relationship between working memory and sleep duration (Banks and Dinges, 2007; del Angel et al., 2015). However, this outcome may be restricted to young adults because a systematic review found that self-reported extreme sleep duration was a risk factor for cognitive aging, including WMC in older adults (Lo et al., 2016).

It is important to explain why nap duration and sleep duration were positively correlated. This outcome is consistent with data demonstrating that self-reported long-duration sleepers reported both longer nocturnal sleep and longer naps compared with normal-duration sleepers (Patel et al., 2012). Interestingly, short-duration sleep may be of more concern than long-duration sleep among university students (Steptoe et al., 2006). Accordingly, it is not surprising that longer nocturnal sleep duration was related to longer nap duration in young adults.

Finally, our study has some limitations. We used a cross-sectional design; thus, we were not able to establish causal relationships between the independent variables and WMC. It was not possible to determine the number of potential subjects who were excluded or who dropped out during the various stages of the recruitment process because we did not have access to the informal preliminary contacts and were not privy to the reasons why particular subjects were excluded. Furthermore, our subjects were young, healthy, highly educated university students; thus, our findings may not be generalizable to populations with a different educational background. This limitation is inherent in investigations using a cohort of college students (Song et al., 2008; Jung et al., 2010; Takeuchi et al., 2010). Thus, studies using larger and more representative samples are needed to confirm our results. Despite these limitations, our study furthers understanding of the association between sleep habits and WMC and provides insights for improving working memory by controlling sleep habits in individuals with impaired working memory and learning.

## Author Contributions

SN, HT, YT, and RK designed the study. SN, HT, RN, AS, YK, CM, KI, RY, TS, YY, SH, TA, KK, and YS collected the data. SN and HT analyzed the data and prepared the manuscript. All authors reviewed the manuscript.

## Conflict of Interest Statement

The authors declare that the research was conducted in the absence of any commercial or financial relationships that could be construed as a potential conflict of interest.

## Footnotes

1 http://www.health.ihe.tohoku.ac.jp/eng_front/medical_checkup/
